# The complete chloroplast genome sequence of *Viola prionantha* (Violaceae)

**DOI:** 10.1080/23802359.2020.1792370

**Published:** 2020-07-22

**Authors:** Chunyan Duan, Kay Zhang, YiZhong Duan

**Affiliations:** a Agricultural College, Henan University of Science and Technology, Luoyang, China; b College of Life Sciences, Yulin University, Yulin, China

**Keywords:** Violaceae, *Viola prionantha*, chloroplast genome, phylogenetic

## Abstract

*Viola prionantha* belongs to the family Violaceae. It has been widely used for a traditional Chinese herb in China. We determined the complete chloroplast genome sequence of *V. prionantha*. The whole chloroplast genome was 156,501 bp in length, consisting of a pair of inverted repeats (IR) of 26,404 bp, each, a large single-copy (LSC) region of 85,689 bp, and a small single-copy (SSC) region of 18,004 bp. We annotated 131 genes, including 84 coding sequences, 8 rRNA sequences, 37 tRNA sequences, and 2 pesudogenes. Among the annotated genes Phylogenetic analysis revealed that *V. prionantha* and *Viola seoulensis* clustered together as sisters.

*Viola prionantha* is a perennial herb that belongs to the genus *Viola* in the family Violaceae, distributed in China, North Korea, and Siberia (Duan et al. 2004; Wang 2009; Cheon et al. 2019; Kyeong et al. 2020). *Viola prionantha* has ornamental value and medicinal value with antibacterial activity (Liu and Sun 2006; Zhou 2008). *Viola* L. is known as one of the more difficult groups to classify. The phylogenetic relationships are still unclear among the genus (Yockteng et al. 2003; Liang and Xing 2010). In recent years, some studies have been conducted on the resources, medicinal properties, physiological characteristics, tissue culture *in vitro*, plant regeneration and heavy metal enrichments of the species (Zhang et al. 2012, 2013; Li et al. 2015; Zhao et al. 2016), but there have been no reports on the whole chloroplast genome of it. cpDNA (chloroplast DNA) is present in the mesophyll cells of green plants. In this study, we sequenced, assembled, annotated the chloroplast genome for further studies on the phylogenomics of *V. prionantha.*

The sampled *V. prionantha* fresh leaves were collected from Luoyang (34°64039.100N, 112°38080.700E), Henan province, in China. A voucher specimen (no. haust69l09) was deposited in the Henan University of Science and Technology herbarium. The total DNA was extracted according to a modified CTAB method (Doyle and Doyle 1987). The extracted genomic DNA was used for sequencing with the Illumina NovaSeq platform. The reference genome of *Viola seoulensis* (GenBank accession number: KP749924) and the programs such as SPAdes (Bankevich et al. 2012) and CpGAVAS (Liu et al. 2012) were used for sequence assembly and annotation. The cpDNA physical map was drawn using the OGDRAW tool (Greiner et al. 2019). Moreover, the complete chloroplast genome sequence was deposited in the GenBank database and a phylogenetic tree was constructed.

The plastid genome of *V. prionantha* (GenBank accession no. MT610374) forms a circular structure comprising 156,501 bp in length with 36.29% GC content, consisting of a pair of inverted repeats (IR) of 26,404 bp, each, a large single-copy (LSC) region of 85,689 bp, and a small single-copy (SSC) region of 18,004 bp. We annotated 131 genes, which consisted of 84 coding sequences, 2 pesudogenes, 8 rRNAs, and 37 tRNAs. Among the annotated genes, 17 genes contain one or two introns. we conducted a phylogenetic analysis. A phylogenetic tree was constructed based on the following chloroplast genomes (accession number in parentheses) (Figure 1). The genome sequences were aligned with MAFFTv7.427 (Katoh and Standley 2013) and then the maximum-likelihood (ML) tree was conducted using RAxMLv.8.2.10 with 1000 boot-strap replicates and the GTRGAMMA model (Stamatakis 2006). The phylogenetic analysis indicated *V. prionantha* was closely related to *V. seoulensis.*

**Figure 1.**
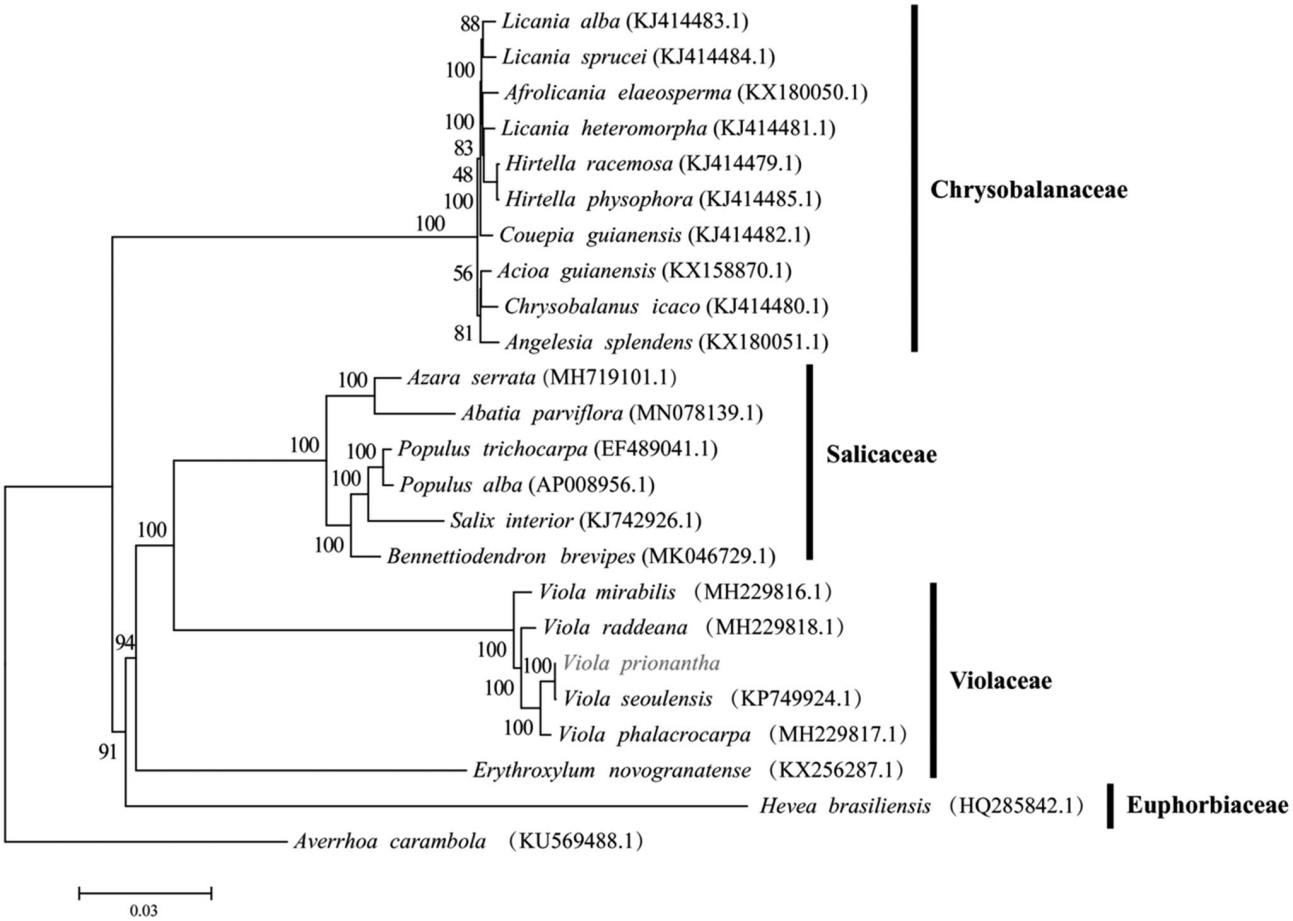
The ML tree using RAxML v.8.2.10 with the GTRGAMMA model.

## Data Availability

The data that support the findings of this study are openly available in NCBI (the National Center for Biotechnology Information) at https://www.ncbi.nlm.nih.gov/, reference number [MT610374], or available from the corresponding author.
